# Super-Resolution Swin Transformer and Attention Network for Medical CT Imaging

**DOI:** 10.1155/2022/4431536

**Published:** 2022-12-08

**Authors:** Jianhua Hu, Shuzhao Zheng, Bo Wang, Guixiang Luo, WoQing Huang, Jun Zhang

**Affiliations:** ^1^Computer Engineering Technical College, Guangdong Polytechnic of Science and Technology, Zhuhai Guangdong, China; ^2^School of Computer Science, Guangdong Polytechnic Normal University, Guangzhou Guangdong, China

## Abstract

Computerized tomography (CT) is widely used for clinical screening and treatment planning. In this study, we aimed to reduce X-ray radiation and achieve high-quality CT imaging by using low-intensity X-rays because CT radiation is damaging to the human body. An innovative vision transformer for medical image super-resolution (SR) is applied to establish a high-definition image target. To achieve this, we proposed a method called swin transformer and attention network (STAN) that uses the swin transformer network, which employs an attention method to overcome the long-range dependency difficulties encountered in CNNs and RNNs to enhance and restore the quality of medical CT images. We adopted the peak signal-to-noise ratio for performance comparison with other mainstream SR reconstruction models used in medical CT imaging. Experimental results revealed that the proposed STAN model yields superior medical CT imaging results than the existing SR techniques based on CNNs. The proposed STAN model employs a self-attention mechanism to more effectively extract critical features and long-range information, hence enhancing the quality of medical CT image reconstruction.

## 1. Introduction

Computerized tomography (CT) images are used by doctors in clinical practice to judge a patient's condition. Good image quality is crucial for effective and accurate screening and diagnosis of a patient's condition [1]. CT imaging played a vital role in the diagnosis and treatment of COVID-19 [2–4]. CT images are obtained using X-rays. However, X-ray radiation is harmful to the human body. Therefore, to reduce the auxiliary radiation, the X-ray intensity is reduced during operation, resulting in low resolution and blurring of CT images. Therefore, how to obtain high-definition medical CT images through superresolution (SR) is an important research topic. For high-quality medical CT images, plenty of deep learning- (DL-) based SR techniques have been proposed [4–6].

Convolutional neural networks (CNNs) have been used to accomplish SR tasks. Initially, the SRCNN network and CNN were used for performing high-resolution reconstruction tasks [7]. This is the earliest reconstruction from low-resolution to high-resolution images by using CNNs and point-to-point nonlinear feature mapping and reconstruction. Currently, DL is widely used in SR applications [8–10]. Shan et al. [11] improved the initial CNN-based SR method by introducing residual learning and attention mechanism.

Powerful reconstruction algorithms have been proposed to improve SR capability. In 2016, FSRCNN [12] was proposed to improve the SRCNN model, and upsampling was performed to increase the running speed. Many CNN-based SR algorithms have been proposed to improve residual learning, attention mechanism, model depth, speed, complexity reduction, and SR performance [13–22].

CNNs are used in the mainstream medical CT image SR algorithms because they provide a very high-performance advantage for the image domain. However, CNNs cannot realize long-range feature extraction. The transformer is mostly employed in the audio industry, but it has been, recently, employed for SR as a replacement for CNNs [23, 24] because the transformer can support long-range feature extraction by using a self-attention (SA) mechanism and yields very good performance in the image domain. Transformer models in the field of medical imaging have been extensively studied [25]. The transformer network and the shared attention approach to limit feature extraction improve the SR performance. Nevertheless, few studies have used the transformer network to improve the SR of medical images. As such, in this study, we attempted to use the transformer network for SR reconstruction of medical CT images.

To reconstruct medical CT images, in this study, we developed the swin transformer and attention network (STAN) model. The main advantage of STAN is that it can learn feature information better. STAN consists of three types of blocks: the low-frequency feature extraction block, deep feature extraction block (including attention transformer blocks (ATBs)), and high-resolution image reconstruction block.

To preserve the low-frequency information, the low-frequency feature extraction block is directly connected to the reconstructed model. The deep feature extraction module mainly consists of ATBs. To extract image edge and texture information, a shift window size is used, which reduces resource consumption. Finally, in the high-resolution image reconstruction block, the features of the first two models are first obtained, multilayer feature fusion is performed, and finally, low-resolution to high-resolution reconstruction is realized.

The main contributions of this study are as follows:

1. A swin transformer is proposed in this paper for an SR network of medical CT images. The use of the attention mechanism improves the network's ability to extract features and edge and flat area information from medical CT images and reconstruct high-quality CT images
2. We developed a low-frequency extraction module with an attention mechanism to capture the long-range dependency feature of the image
3. To handle long-range dependency images, we used a shift window mechanism, overcoming the traditional transformation limitation of dividing the input image into fixed-size patches

## 2. Related Work

The traditional SR algorithm uses a bicubic interpolation algorithm to upsample an image and has the disadvantages of losing details and blurring the image. Therefore, neural networks have been employed for SR. The transformer network can further improve the performance of a traditional CNN. With the development of SR, many scholars have applied the SR technology to improve the clarity and reliability of medical CT images by employing the following three approaches:

1. Obtaining SR images by using CNNs: CNNs are mainly used to perform transformations between images of different resolutions (e.g., LR image to HR image). Due to the different characteristics of the image, different image scaling methods need to be used to recover different image details. Therefore, nonlinear mapping is performed to recover the lost high-frequency details. CNNs are widely used to reconstruct high-quality images and realize SR through dense connection convolution, multichannel networks, and symmetric jump connections [26, 27]
2. The use of transformer networks in the field of image applications: transformer networks are generally used in the audio field. Because of their local attention mechanism and long-term compliance, transformer networks are highly suitable for image feature extraction. Therefore, the transformer [28, 29] is widely used in the field of image processing because of its ability to better access information and integrate the CNN and transformer. Pan et al. [23] proposed a high-quality reconstruction transformer to capture image global features for medical CT image reconstruction
3. The use of SR in the field of medical CT imaging: DL technology is extensively employed for medical CT imaging [30–32]. Many scholars have applied SR technology to the medical field [33–35]. SR technology is used to reconstruct high-definition images for the characteristics of medical images, which can effectively improve image quality and reduce X-ray radiation to the human body

In this study, we designed the STAN model to reconstruct medical CT images. In addition, we introduced a self-information mechanism in the network model to enable updates to be performed on long-range information; moreover, the medical image quality smoothing area enables better image quality.

## 3. Methods

The architecture of the medical CT image performance enhancement network is presented here. For the SR reconstruction of medical CT images, a transformer and an attention network are employed. To improve the extraction of low-frequency and high-frequency medical feature information, we designed the STAN model. We used a transformer network instead of traditional CNNs to considerably increase the quality of medical CT images and edge information. The proposed system comprises three types of blocks: low-frequency feature extraction block, deep feature extraction block, and high-resolution image reconstruction block.

### 3.1. Network Architecture

**Figure 1:**
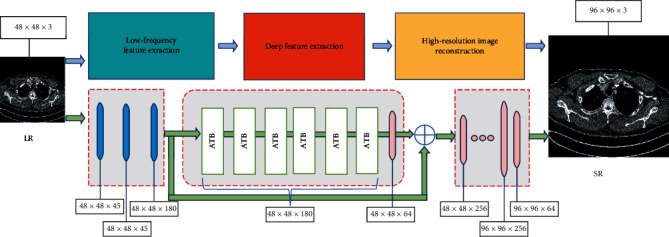
The architecture of the proposed STAN model. The STAN includes the low-frequency feature extraction block (for the extraction of flat area image information), deep feature extraction block (including six ATBs), and high-resolution CT image reconstruction block (for the feature extraction of image edge information).

The structure of the proposed STAN model is illustrated in Figure 1. STAN employs an efficient long-range attention transformer network for reconstructing high-resolution images from low-resolution medical CT images. STAN includes the low-frequency feature extraction block (for the extraction of flat area image information), deep feature extraction block (including six ATBs), and high-resolution CT image reconstruction block (for the feature extraction of image edge information). Low-resolution images are inputted into the STAN model. The low-frequency feature extraction block extracts the low-frequency feature information from medical CT images by using multilayer CNNs. The deep feature extraction block employs the self-attention mechanism transformer network to extract the edge information of medical CT images, and multichannel image information is obtained by adding to the previous network. The high-resolution CT image reconstruction block fuses the low-frequency and high-frequency information; in addition, it extracts the characteristic data of multiple channels and upsamples the image to obtain an SR medical CT image.

The proposed STAN algorithm is shown below. The low-resolution input image is *I*_LQ_. Transform *H*_LF_ is a low-frequency feature extraction block. In the deep feature extraction block, there are *M* ATBs, and each ATB has *L* STLs and a convolution operation. After the deep feature extraction block, the high-quality picture is reconstructed through high-resolution CT image reconstruction.

### 3.2. Low-Frequency Feature Extraction Block

**Figure 2:**
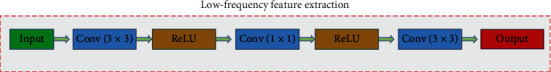
Low-frequency feature extraction block.

As shown in Figure 2, the low-frequency feature extraction block realizes low-frequency information extraction and includes three layers. A low-resolution image is input in. After feature extraction by using 3 × 3 convolution operations, fine feature extraction is performed using 1 × 1 convolution. Finally, the low-frequency information extraction output of the current block is obtained using a convolution kernel size of 3 × 3.

A low-resolution image is input as *I*_LQ_. Then, two 3 × 3 and one 1 × 1 convolution layer are used to obtain the low-frequency feature output as follows:

(1) $$\begin{array}{c} F_{0}=H_{\text{LF}}\left(I_{\text{LQ}}\right). \end{array}$$

This module uses a multilayer network to better accomplish the extraction of low-frequency information.

### 3.3. Deep Feature Extraction Block

Deep features *F*_DF_ ∈ *R*^*H*×*W*×*C*^ are extracted from the low-frequency feature output *F*_0_ as follows:

(2) $$\begin{array}{c} F_{\text{DF}}=H_{\text{DF}}\left(F_{0}\right), \end{array}$$

where comprises six ATBs. The composition and principle of the ATB are described in detail here.

**Figure 3:**
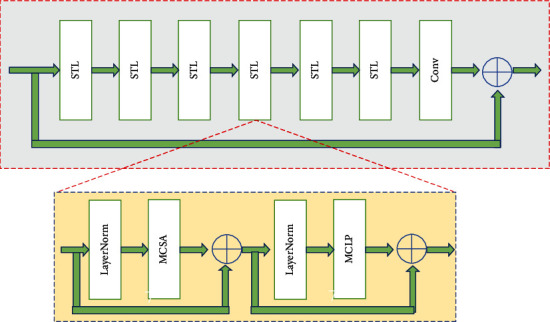
Attention transformer block.

As shown in Figure 3, ATBs are composed of swin transformer layers (STLs) and convolutional layers with self-awareness functions. The STL is the base component of the ATB. The base network comprises multiple STLs and ends with a convolutional layer to form the ATB. In this study, the number of STLs in an ATB was set as 6 to achieve a balance between extraction performance and model complexity.

*F*_*i*,0_ indicates the *i*-th ATB. Information features *F*_*i*,1_, *F*_*i*,2_,…, *F*_*i*,*L*_ are extracted by the ATB layers as follows:

(3) $$\begin{array}{c} F_{i,j}=H_{\text{ST}{\text{L}}_{i,j}}\left(F_{i,j-1}\right),j=1,2,\cdots ,L, \end{array}$$

where *H*_STL_*i*,*j*__(·) is the *i*-th ATB and *J* denotes the *j*-th STL. This design offers two advantages: spatial variation convolution and residual connection reconstruction module.

The output of ATB can be formulated as

(4) $$\begin{array}{c} F_{i,\text{out}}=H_{\text{CON}{\text{V}}_{\text{i}}}\left(F_{i,L}+F_{i,0}\right), \end{array}$$

where *H*_CONV_*i*__(·) is the *i*-th ATB swin transformer.

The STL enables the self-attentive mechanism through the transformer layer. Its most important feature is the use of local attention and shift window mechanism. By the size *H* × *W* × *C*, the ATB splits the input into nonoverlapping *M* × *M* local windows. In this manner, the input size is reshaped into the (*HW*/*M*^2^) × *M*^2^ × *C* feature, where *HW*/*M*^2^ is the number of windows.

The STL consists of three components: layer specification (LN) layer (used for regularization), multicontrol head SA (MCSA) layer, and multilayer control perceptron (MLCP) layer. The MLCP layer is composed of two completely connected neural networks, and feature extraction is performed between them through nonlinear transformation. The LN layer is added before the MCSA and MLCP layers, and then, the residuals are used to connect the two modules. The process is as follows:

(5) $$\begin{array}{c} X=\text{MCSA}\left(LN\left(X\right)\right)+X, \end{array}$$

(6) $$\begin{array}{c} X=\text{MCLP}\left(LN\left(X\right)\right)+X. \end{array}$$

### 3.4. High-Resolution Image Reconstruction Block

The low-frequency feature extraction of high-quality images is performed from medical CT images according to the convergence:

(7) $$\begin{array}{c} I_{\text{RHQ}}=H_{\text{REC}}\left(F_{0}+F_{\text{DF}}\right), \end{array}$$

where *H*_REC_(·) indicates the reconstruction model. The low-frequency information mainly includes low frequencies, while the deep features are used to repair the missing high frequencies. Sloshing inverter circuits are used to transmit low-frequency information to the medical CT image reconstruction module through a high skip connection and help the deep-level feature collection module to focus on high-frequency information.

**Figure 4:**
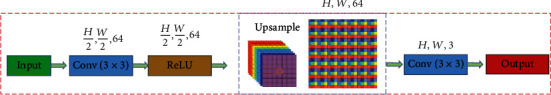
High-resolution image reconstruction blocks.

The high-resolution image reconstruction block (Figure 4) comprises a 64-channel CNN with channel size *H*/2, *w*/2, 64. The 64-channel feature map output is obtained using the pixel shuffle upsampling method. Finally, a 3-channel CNN is used to generate the high-definition image output.

The primary function of pixel shuffle is to convert the multichannel feature map *r*∗*r* into size of *w*∗*r* and *h*∗*r* (e.g., the original feature map size is 4 × 128 × 128, which is then adjusted to size 1∗(128 × 2)∗(128 × 2)), where *r* is the upsampling factor, which is the magnification of the image.

## 4. Results

We evaluated the performance of the proposed model on open-source datasets and evaluated the image quality by using the peak signal-to-noise ratio (PSNR) metric. Compared with other advanced SR methods, the proposed model offers obvious performance advantages.

### 4.1. Dataset

We used the largest medical CT medical image dataset, DeepLesion [36], for training and testing the model. This dataset not only includes key CT slices containing the important lesions but also provides the three-dimensional context (additional slices of 30 mm above and below the key slices). The size of the dataset is 221 GB. Because of the huge amount of data, 11,500 high-quality CT images were randomly selected and divided into three parts. The majority of the images were used for training (10,000), and the remaining were used for verification (1000) and testing (500). This dataset consists of the original image and the downsampled image through bicubic interpolation by using the function torchvision.transforms.resize() in the PyTorch library. The source HR medical CT image was reduced to a LR image as the input data, and the original HR medical CT medical image was used as the data label to be used as the input dataset of the DL neural network for training. For the sake of accuracy of model training, the training set was added through data enhancement to improve the generalization ability.

### 4.2. Implementation Details

The three-channel (RGB) pixels of the input image and the original data were linearly reduced to obtain the LR image, and the original data were used as label data and inputted into the network. Six ATBs were used. The sliding window size of each transform network was set as 8, and the patch size corresponding to the LR image resolution was 48.

Adam optimizer was adopted with two improvements: gradient sliding average and bias correction. The learning rate decayed with each update factor decay set as 0.999, and the initial learning rate was 2 × 10^−4^. The pixel shuffle method was used for image upsampling.

### 4.3. Evaluation Index

We evaluated the reconstructed SR images by using two methods: subjective evaluation and objective evaluation. Many factors influence subjective evaluation, and the reconstructed SR images are evaluated mainly based on human visual perception. In this study, the PSNR was measured as the objective evaluation metric to study the performance of high-resolution restoration networks for medical CT images. To demonstrate the superiority of the proposed model visually, we calculated the PSNR values of the SR images generated using the proposed method and other methods and compared them.

PSNR is an objective criterion for evaluating images. The calculation method is as follows:

(8) $$\begin{array}{c} \text{MSE}=\frac{1}{mn}\overset{m-1}{\underset{i=0}{\sum }}\overset{\text{n}-1}{\underset{j=0}{\sum }}{\left\|J\left(i,j\right)-L\left(i,j\right)\right\|}^{2}. \end{array}$$

Then, PSNR can be obtained as follows:

(9) $$\begin{array}{c} \text{PSNR}=10\log_{10}\frac{\text{MaxValu}{\text{e}}^{2}}{\text{MSE}}=10\log_{10}\frac{2^{\text{bits}}-1}{\text{MSE}}, \end{array}$$

where *J* and *L* are the two pixel values and the size of the image is *m* × *n*. The greater the PSNR, the better the medical CT image effect, and vice versa.

### 4.4. Ablation Study

To better understand how STAN performs SR in medical CT images, a comprehensive ablation study on ATBs was performed to evaluate the role of key parts of the proposal STAN model, as well as the degree of depth and the choice of shared attention mechanism.

As can be seen in Table 1, we studied the effect of the removal and addition of ATB modules on the performance of the medical CT image reconstruction network. To analyze the effect of the low-frequency feature extraction block and ATB on the performance of the STAN model, we conducted ablation experiments by using different numbers of modules and studied their corresponding PSNR performance under the ×4 scaling condition. The number of ATBs affects the PSNR, i.e., the higher the number of ATBs, the higher the PSNR.

**Figure 5:**
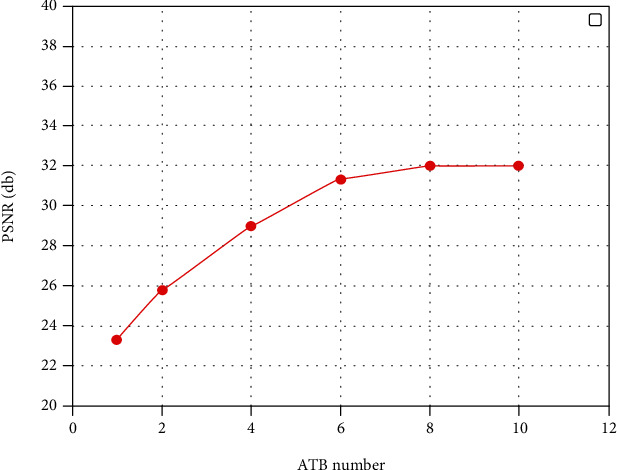
Relationship between PSNR and number of ATBs in STAN.

As shown in Figure 5, we studied the relationship between the number of ATBs and the PSNR performance on DeepLesion for image SR (×4). To obtain a relatively lightweight model, the number of ATBs was selected as 6, and the number of convolutional layers was 3 in the final test performance experiment.

### 4.5. Analysis of Experimental Results

Network optimization was performed. The performance comparison results in terms of PSNR with ×2 and ×4 scale factors are presented in Table 2. We analyzed different algorithms on the DeepLesion testing set. Compared with the bicubic method, the PSNR of STAN improved by 9.58 and 13.36 dB when the scale factor was ×2 and ×4, respectively. Compared with the method using the DL neural network, the PSNR of STAN improved by 3.81 and 3.56 dB when the scale factor was ×2 and ×4, respectively.

**Figure 6:**
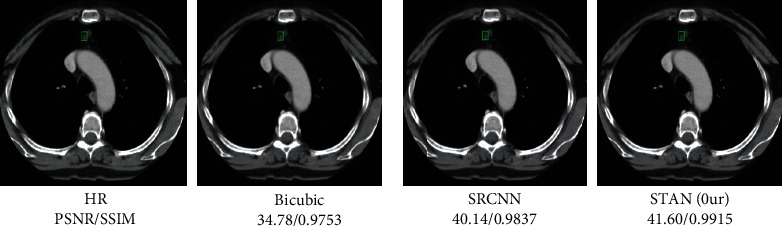
Medical CT image testing using different algorithms on the DeepLesion dataset.

As can be seen in Figure 6, the bicubic reconstruction of medical CT images yielded the worst effect and the lowest PSNR. The SR algorithm based on DL performed better than the algorithm based on interpolation. The STAN model based on transform networks proposed in this paper performed relatively better than the CNN-based SR method by 0.76 and 0.23 dB when the scale factor was ×2 and ×4, respectively.

Thus, the proposed STAN model exhibited superior performance to the CNN-based SR method, demonstrating that the transformer network yields obvious performance advantages in medical CT imaging.

By using different algorithms, the medical CT image was reconstructed with multiple resolutions. The results of different algorithms on the DeepLesion are shown in Figure 6.

## 5. Conclusions

For SR of medical CT images, we proposed an improved STAN model that uses the SA mechanism for feature extraction and solves the long-range dependency problem encountered in CNNs and RNNs. In addition, it can obtain more important feature information. In STAN, nonoverlapping feature values are computed using different window sizes, and feature extraction is performed using a shared-attention mechanism.

We experimentally demonstrated the SR effectiveness of the proposed STAN model in medical CT images. We used the PSNR metric for performance comparison. The results revealed that the PSNR of the proposed STAN model is much better than that of the CNN SR method. The use of the SA mechanism in STAN yields clearer reconstruction results, and the reconstruction effect in the low-frequency regions of medical CT images is better. However, medical imaging may generate image noise due to the influence of hardware equipment and the external environment. As such, the next step is to denoise medical CT images in the SR process.

## Figures and Tables

**Algorithm 1:**
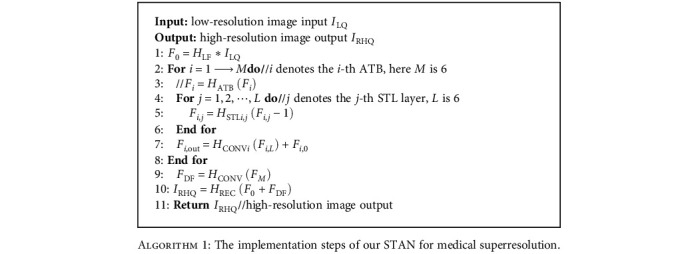
The implementation steps of our STAN for medical superresolution.

**Table 1:** Ablation study on ATB design.

ATB number		
Number of ATBs	X4	PSNR
1	√	23.3
2	√	25.78
4	√	28.967
6	√	31.34
8	√	31.90
10	√	32.02

**Table 2:** PSNR (dB) values for the proposed STAN model.

Model	×2	×4
Bicubic	23.32	21.76
SRCNN [37]	33.17	27.78
DRRN [38]	34.56	29.65
MDSR [39]	34.85	29.90
RDN [40]	34.96	30.24
RCAN [41]	34.99	30.35
LTE [42]	36.22	31.11
STAN (our)	36.98	31.34

## Data Availability

The medical CT medical image data used to support the findings of this study have been deposited in the https://nihcc.app.box.com/v/DeepLesion repository. This is an open source medical open source dataset, you can download and use it freely.
